# A Rare Case of Spontaneous Intussusception in an Elderly Patient

**DOI:** 10.7759/cureus.40726

**Published:** 2023-06-21

**Authors:** Falgun Gosai, Kyle Espejo, Rachel Zussman, Stephen DeMartini, Chandler Wilfong

**Affiliations:** 1 Hospitalist, Saint Francis Medical Center, Peoria, USA; 2 Medicine, University of Illinois College of Medicine Peoria, Peoria, USA; 3 Surgery, Saint Francis Medical Center, Peoria, USA

**Keywords:** currant jelly stool, elderly population, elderly, lead point, intussusception

## Abstract

Intussusception is an uncommon pathology in the adult population. Most intussusception cases result from an underlying pathological lead point, oftentimes a malignant neoplasm. We report a case of intussusception in an adult male patient who presented with abdominal pain and currant jelly diarrhea. The patient underwent laparoscopic right hemicolectomy and the biopsy of the affected colon did not show any pathological lead point. Intussusception remains an important differential diagnosis in patients presenting with abdominal pain and bloody diarrhea.

## Introduction

Intussusception describes the invagination or “telescoping” of one segment of the bowel into an adjacent segment, with the potential to lead to bowel obstruction, ischemia, and necrosis [1]. While intussusception is a common pediatric pathology and often idiopathic, intussusception in adults is rare and nearly always occurs due to an underlying pathological lead point [1,2]. During peristalsis, the lead point becomes trapped in a distal segment of the bowel, causing the bowel to telescope into itself [2]. It is estimated that adults account for less than 5% of all cases of intussusception, with 77% of these cases caused by an underlying malignant neoplasm [3,4]. Other causes of intussusception include polyps, lipomas, Meckel’s diverticulum, and Peutz-Jeghers syndrome [5]. Here, we report a case of intussusception in a 61-year-old male with no underlying pathological lead point, highlighting the importance of intussusception remaining on the differential for adult patients presenting with abdominal pain.

## Case presentation

**Figure 1:**
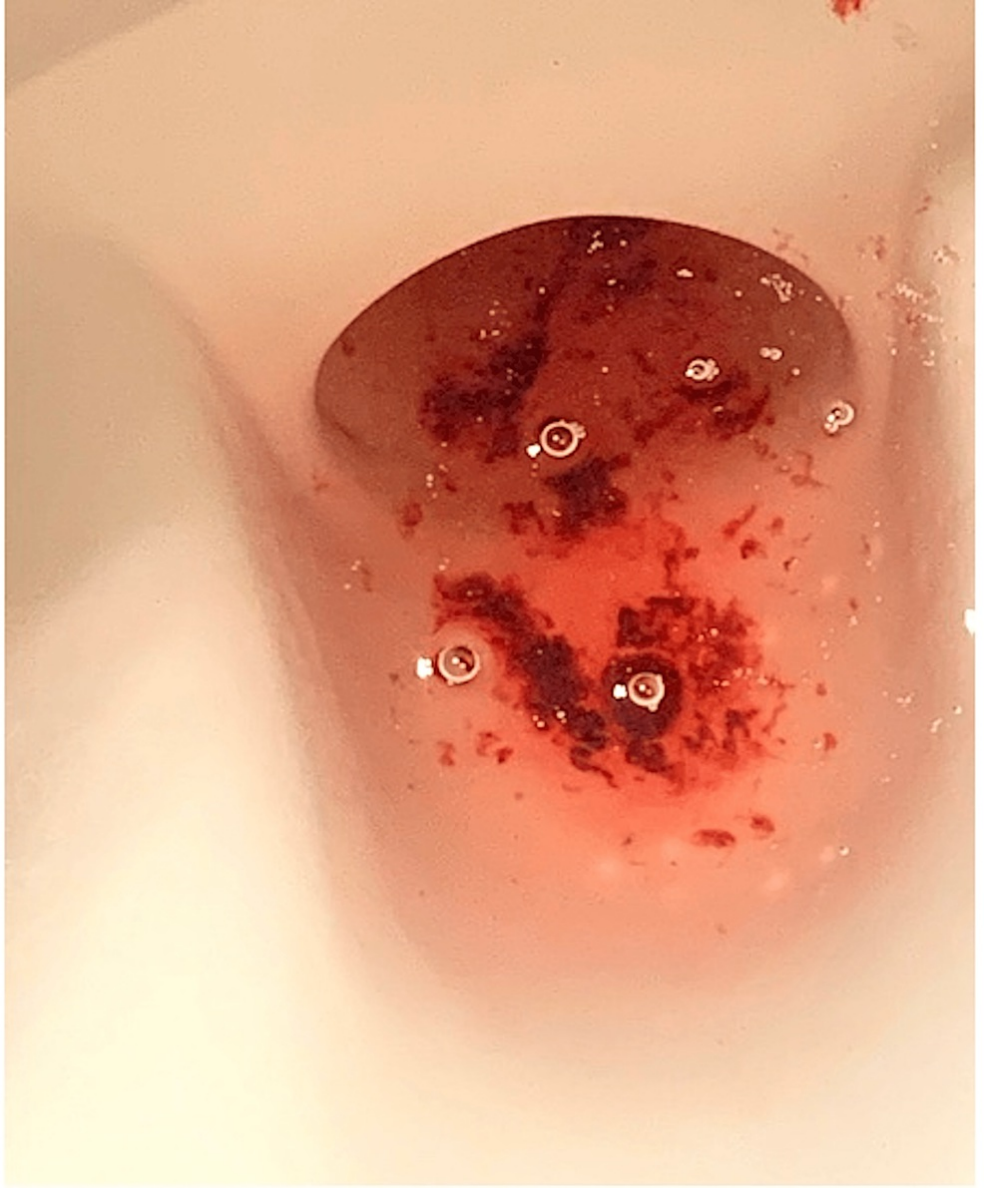
Currant jelly stool, a common presentation of intussusception.

A 61-year-old male presented to the emergency department from an urgent care center with a one-day history of diffuse, intermittent, crampy abdominal pain, nausea, and poor appetite. The abdominal pain had no relation with food intake. He also had seven to eight episodes of red, currant jelly diarrhea (Figure 1). He did not have fever, chills, or vomiting. The patient’s past medical history included type 2 diabetes mellitus on treatment with metformin. His surgical and family history were unremarkable. He had a history of tobacco intake 27 years ago. Vital signs were unremarkable except tachycardia (110 beats/minute). Physical examination showed mild tenderness over the right lower abdomen without guarding or rigidity.

**Figure 2:**
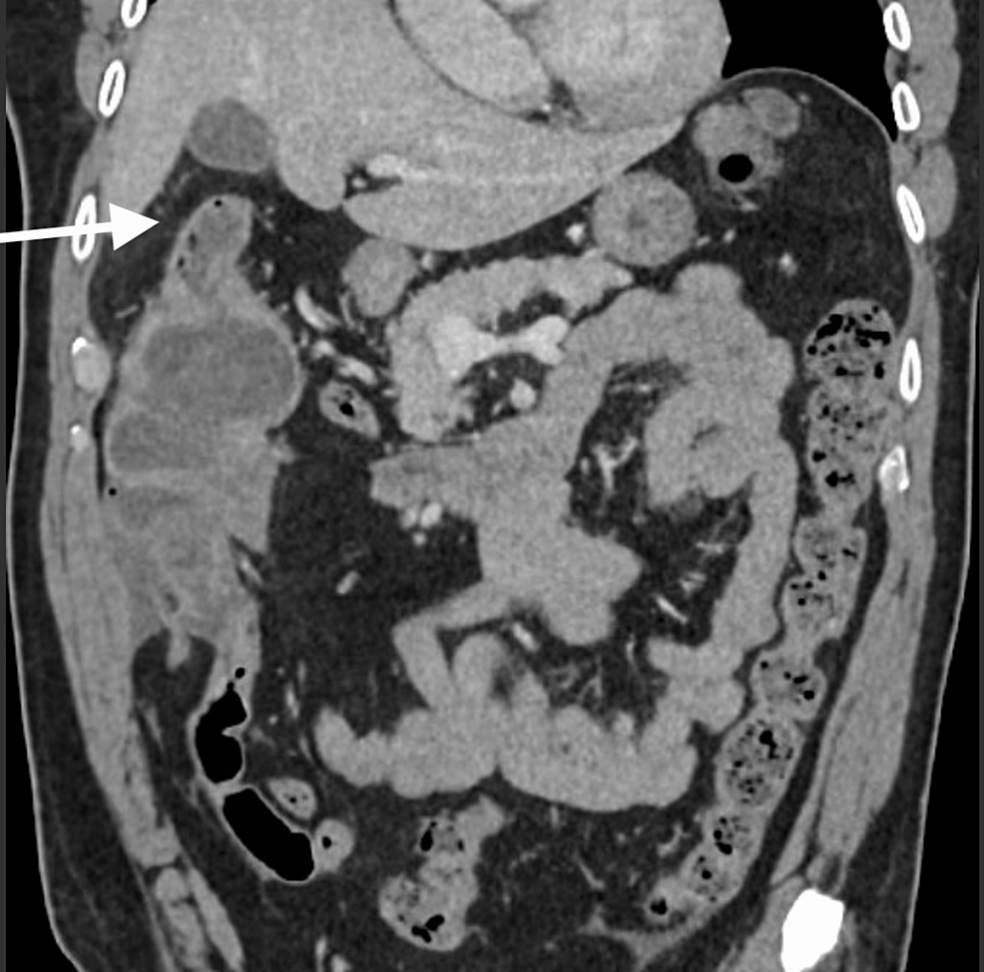
CT of the abdomen shows intraluminal hemorrhage or a mass and abrupt bowel caliber change in the vicinity of the hepatic flexure. The white arrow: an abrupt bowel caliber change in the vicinity of the hepatic flexure.

The complete blood count was within normal limits. The comprehensive metabolic panel showed a glucose level of 142 mg/dL and a blood urea nitrogen/creatinine ratio of 25:1 mg/dL. Two sets of blood cultures were negative. CT of the abdomen/pelvis with oral and intravenous contrast demonstrated diffuse mural wall thickening and enhancement of the ascending colon, with possible intraluminal hemorrhage or a mass and abrupt bowel caliber change in the vicinity of the hepatic flexure (Figure 2). There was a suspicion of intussusception of the cecum into the ascending colon. No lymphadenopathy was noted in the ileocecal region.

**Figure 3:**
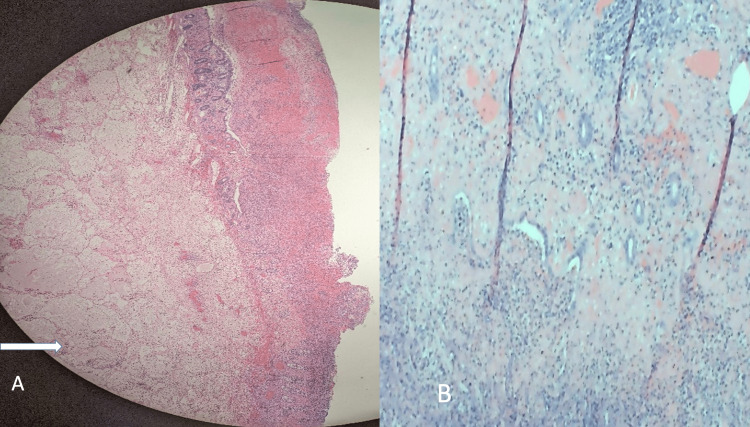
The biopsy of the resected edematous colon showing diffuse edema and inflammation suggestive of non-viability and ischemia. The white arrow in A shows diffuse edema. B shows neutrophilic infiltration.

The patient was made NPO (nil per oral) and a nasogastric tube was placed draining 800 cc of bilious fluid. The patient underwent immediate laparoscopic hand-assisted right hemicolectomy (laparoscopy images not available). There was no gross evidence of metastasis. The right colon was mass-like, inflamed, and found to be intussuscepting into itself. The cecum was intussuscepted into the ascending colon. After a right hemicolectomy, the terminal ileum and proximal transverse colon were anastomosed in a side-to-side fashion. The portion of the folded colon was biopsied. The pathology of the biopsy showed mucosal non-viability with inflammation, compatible with the appearance of colonic intussusception (Figure 3).

The postoperative course was unremarkable. The patient had normal bowel function the next day and tolerated a general diet. He was discharged on postoperative day three on a high-fiber diet with plans to pursue a colonoscopy in three to four months.

## Discussion

Intussusception, a common cause of intestinal obstruction in children, is a very rare cause of mechanical bowel obstruction in adults. The incidence in adults is approximated as 2-3 cases/1,000,000 population per year [6]. Overall, 95% of intussusception cases in children are idiopathic in nature, whereas 80-95% of intussusception cases in adults are due to an identifiable cause such as malignancy, postoperative adhesions, inflammatory bowel disease, intestinal tubes, jejunostomy feeding tubes, or prior gastric surgery [6,7]. In a small retrospective study of adults with intussusception, 12.5% of patients had no definable lesion causing their intussusception [8]. This case describes an adult male patient with intussusception, likely idiopathic in nature due to the absence of an identifiable pathological lead point. The patient did not have any previous abdominal surgery.

A majority of cases of intussusception may not have red currant jelly stools on presentation. The CT is useful in the diagnosis of intussusception and associated complications [9]. The intussuscepted bowel among children can be approximated as a conservative treatment [10]. Surgical intervention is often the most appropriate course of action among adults due to its association with an underlying malignancy. Small bowel intussusceptions are managed with laparoscopic exploration and reduction, while large bowel involvement may need surgical correction [5,11].

We demonstrate the utility of keeping intussusception on the differential for acute abdominal pain in combination with appropriate imaging. Patients presenting with abdominal pain are at risk of repeat visits and misdiagnosis, especially in resource-limited settings where the availability of CT scans is limited [12]. This case in particular was diagnosed quickly due to a timely imaging workup.

## Conclusions

Intussusception in adults is a rare diagnosis that often presents with symptoms of abdominal pain, blood in stool, and/or nausea. The vague clinical presentation and low prevalence in adults make the diagnosis challenging, especially in resource-limited settings. High suspicion and timely imaging can avoid the delay in the diagnosis and complications.
